# Visualizing nanostructures in supramolecular hydrogels: a correlative study using confocal and cryogenic scanning electron microscopy

**DOI:** 10.3762/bjnano.16.156

**Published:** 2025-12-12

**Authors:** Shaun M Smith, Ferdinando Malagreca, Jacqueline Hicks, Giuseppe Mantovani, David B Amabilino, Christopher Parmenter, Lluïsa Pérez-García

**Affiliations:** 1 School of Chemistry, GSK Carbon Neutral Laboratories for Sustainable Chemistry, University of Nottingham, Triumph Road, NG7 2TU, United Kingdom; 2 Nanoscale and Microscale Research Centre (nmRC), Cripps South Building (Building 53), University Park, NG7 2RD, United Kingdom; 3 University of Nottingham, School of Pharmacy, NG7 2RD, United Kingdom; 4 Institut de Ciència de Materials de Barcelona (ICMAB-CSIC), Carrer dels Til·lers, Bellaterra, 08193, Spain; 5 Departament de Farmacologia, Toxicologia i Química Terapèutica, Facultat de Farmàcia i Ciències de l’Alimentació, Avda. Joan XXIII 27-31, Universitat de Barcelona, Barcelona, 08028, Spain; 6 Institut de Nanociència i Nanotecnologia IN2UB, Universitat de Barcelona, Barcelona, 08028, Spain

**Keywords:** anion binding, colloid, fluorophore, microscopies, nanostructure, supramolecular hydrogel

## Abstract

Solvated supramolecular hydrogels present unique challenges in nanoscale morphological characterization because of their fragile fibrous nature and low concentration of the solid component. In this study, imidazolium-based hydrogels containing either diketopyrrolopyrrole (DPP) or zinc(II) phthalocyanine (ZnPc) fluorophores were imaged using confocal laser scanning microscopy (CLSM) of fully solvated gels and cryogenic scanning electron microscopy (cryo-SEM) was used to observe the corresponding xerogels. The DPP@Gel systems exhibit strong fluorescence and are effectively imaged using CLSM, with fibre morphologies that closely correlate with those seen with cryo-SEM. In contrast, the analogous imidazolium gel system containing a sulfonated zinc phthalocyanine (ZnPc@Gel) yields poor CLSM images because of the relatively weak emission and sample disruption during compression, whereas cryo-SEM enables clear visualization of the native fibrous network. These results demonstrate the complementary nature of CLSM and cryo-SEM and highlight the value of cryo-SEM as a very useful tool for imaging soft nanomaterials with low fluorescence or limited optical contrast.

## Introduction

Hydrogels, whether based on self-assembling molecules or cross-linked polymers, are useful in fields ranging from tissue engineering to drug delivery and biosensing [1–5]. Their high water content and soft, porous structure make them ideal for mimicking biological environments, yet these same properties pose major challenges for morphological characterization [6–7]. In particular, conventional electron microscopy often requires dehydration, which risks collapsing the delicate network, while optical methods are typically diffraction-limited, preventing direct visualization of nanoscale features [6,8].

Confocal fluorescence microscopy addresses some of these limitations by allowing hydrogels to be imaged in situ, fully hydrated, and often in real time when a fluorophore is incorporated into the colloidal network [2,9]. Through selective incorporation of fluorophores, it is possible to highlight different components of the hydrogel or embedded cells, facilitating 3D reconstruction of the microarchitecture [10]. However, the achievable resolution in conventional confocal microscopy is still restricted to over one hundred nanometres [11].

Cryogenic scanning electron microscopy (cryo-SEM) bypasses many of these resolution limits by preserving the hydrogel through rapid freezing and subsequent fracture, thereby maintaining native-like morphology in the microscope in the form of a xerogel (no solvent) nature [12–14]. High-resolution images of the fibrillar networks can be obtained at a resolution of tens of nanometres or better, revealing fine structural details such as individual fibrils and nanoscale pore walls [6,12,14]. However, the specialized sample preparation required for cryo-SEM (including vitrification, sublimation, and sputter coating) can introduce artefacts if not carefully optimized [15]. Refining cryo-SEM protocols to mitigate freezing artefacts and capture near-native hydrogel features has been carried out to counter these potential anomalies [16].

Complementary studies of soft materials using various forms of microscopy have naturally been undertaken, as comprehensive characterization of such systems requires the application of multiple imaging methods. Within the realm of hydrogels, it is typical to see comparative fluorescence and electron microscopy imaging being used to characterize a given material. The application of confocal microscopy, stimulated emission depletion (STED) microscopy, and conventional transmission electron microscopy (TEM) to a supramolecular peptide material has been shown to allow for sub-diffraction resolution imaging of fibre morphology in situ without covalent modification being required for fluorescence imaging [17]. Similar studies of swollen hydrogels using cryo-SEM, environmental SEM (ESEM), confocal, and light microscopy have revealed that cryo-SEM may introduce morphological artefacts that are not observed in hydrated-state imaging. The presence of these artefacts stresses the need for multiple orthogonal approaches when interpreting gel morphology [18].

**Figure 1:**
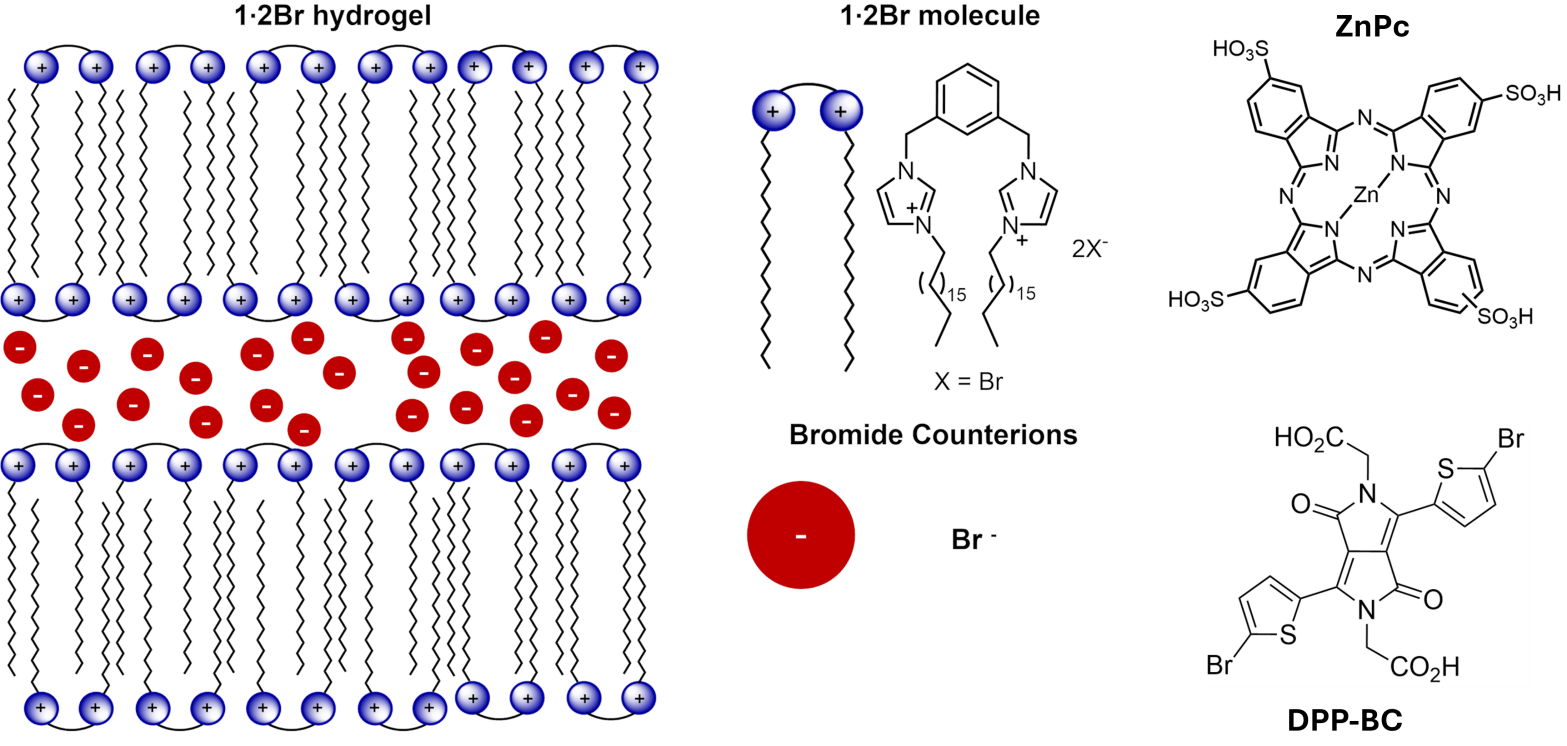
A representation of the fibre structure of the hydrogel that forms upon self-assembly of **1**·2Br in the presence of 1:1 water–ethanol. Alkyl chains form a lamellar structure and between cationic imidazolium layers lie the bromide counterions. Note: sizes are not to scale. The molecular structures of the fluorophores (ZnPc and DPP-BC) are also shown.

In this study, the morphological features of imidazolium-based supramolecular hydrogel fibres incorporating two distinct fluorescent probes, namely, a zinc phthalocyanine tetrasulfonic acid (ZnPc) and a diketopyrrolopyrrole bis(carboxylic acid) (DPP-BC) (Figure 1), are probed using confocal fluorescence microscopy and cryo-SEM. ZnPc is a water-soluble fluorophore with a conjugated and mainly flat macrocyclic structure, characterized by strong absorption in the red region of the visible spectrum, and has been previously studied within imidazolium-based gel system [19] without the methods employed here. The DPP derivative, by contrast, is a water-soluble diketopyrrolopyrrole featuring a central 2,5-dihydro-2,5-dioxo-pyrrolo[3,4-*c*]pyrrole core flanked at the 3- and 6-positions by thiophene rings. The lactam nitrogen atoms are functionalized with carboxymethyl groups, providing aqueous solubility and enabling favourable interactions with the cationic imidazolium-based moieties in the gel network. Together, these probes enable complementary insights into the gel network’s morphology and probe–gel interactions. The comparison is important because it could reveal an effect of the probe on the morphology of the network of fibres. The gel network itself is formed by the self-assembly of 1,3-bis[(3-octadecyl-1-imidazolium)methyl]benzene dibromide (**1**·2Br) in a 1:1 water–ethanol solvent system [20–21] and is made up at the microscale of an entangled fibrous network.

The reliable imaging of these networks containing the bis(imidazolium)-based gel systems is of interest because of the dependence of properties such as drug release [22–24], photodynamic therapy [25], photoreactivity [26], additive manufacturing [27], and mechanical photoresponsiveness [21] on the morphology, and the potential to correlate with confidence between morphology and these and other properties.

## Results and Discussion

### Confocal imaging

#### ZnPc@Gel

**Figure 2:**
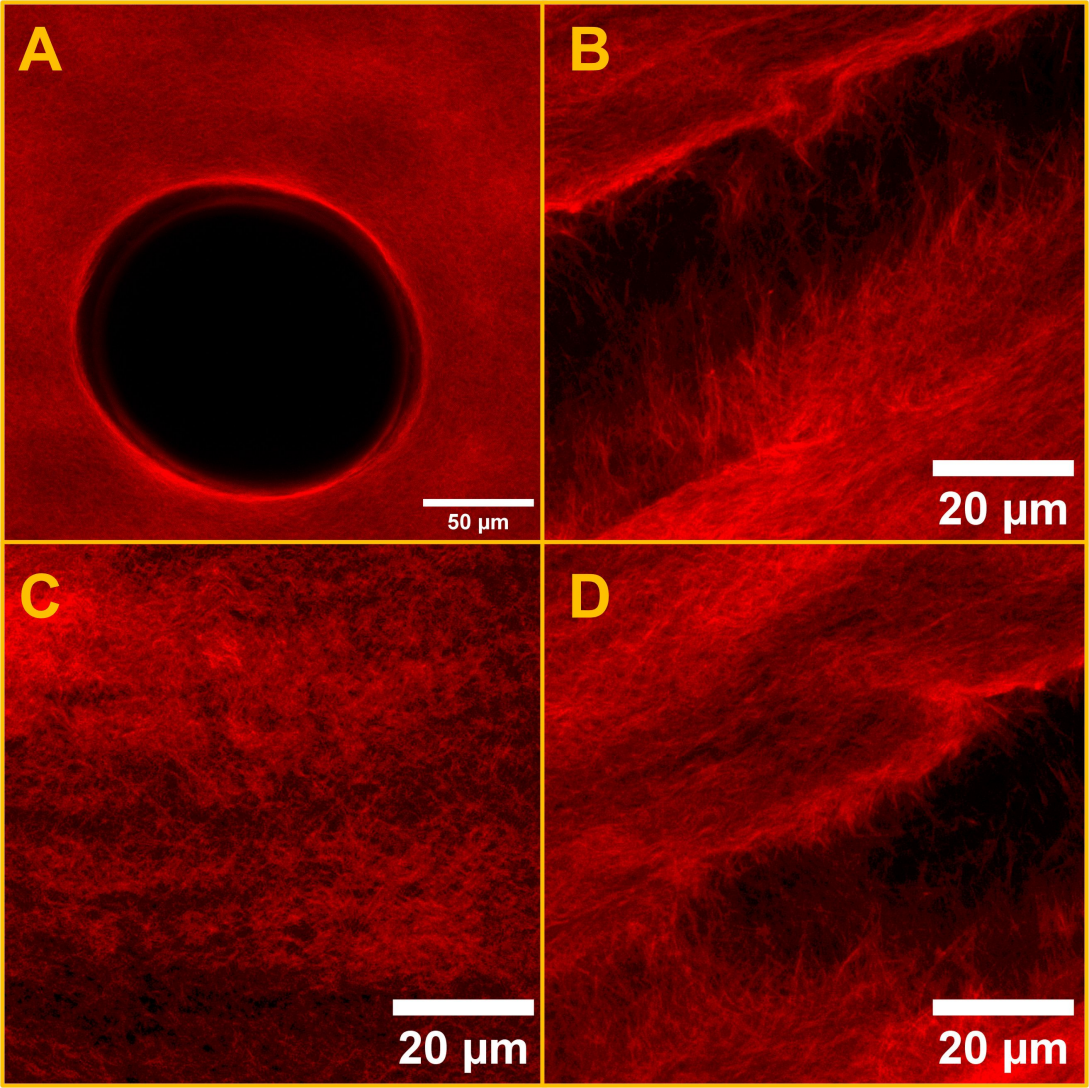
CLSM micrographs of ZnPc@Gel at two magnifications. (A) Micrograph of an air bubble present in a ZnPc@Gel sample. (B) Region where the gel has been pulled apart because of compression of the sample. (C) Homogeneous, unbroken area of ZnPc@Gel. (D) Area exhibiting partial gel disruption. Imaging parameters: laser 650 nm, emission LP filter 650 nm, laser power 100%.

The attempts at using CLSM to image the hydrogels containing ZnPc met with significant challenges in resolving detailed fibre morphology unless the gel was heavily loaded with the fluorophore. In these experiments, ZnPc concentrations exceeding 150 μM were needed to afford a sufficiently detectable fluorescence signal under the confocal conditions employed here (see Experimental section), and even then, excitation laser intensities had to be operated at or near their maximum fluence to obtain images with good signal-to-noise ratio. This elevated laser power then contributed to a certain extent of fluorophore bleaching, making effective imaging even more challenging. Despite these measures, the resulting micrographs largely depicted aggregated domains or regions of mechanical disruption (Figure 2A–D), where the gel network had broken during sample compression between glass slides or where air bubbles were trapped (Figure 2A). In comparatively intact regions of the gels, fibrillar features were visible but appeared diffuse and poorly resolved, indicating that the low fluorescence quantum yield of ZnPc (see Supporting Information File 1) did not allow CLSM to provide high-contrast, high-resolution imaging of the gel’s internal superstructure.

The intrinsic weak emission of ZnPc not only necessitated the application of high excitation powers but also required physical compression of the gel sample to reduce path length and thus improve the signal-to-noise ratio. However, this approach introduced the evident result of mechanical artefacts, such as gel network disruption or the artificial formation of gaps and separated fibre bundles. The clearest fibrillar structures were often only observable in regions where the gel had fractured under compression, further complicating any interpretation of what might be the true native morphology. Increasing the ZnPc loading beyond 150 μM failed to significantly enhance the resolution of fibrillar features, suggesting that limitations stemmed primarily from the photophysical properties of the fluorophore rather than from concentration-dependent effects alone.

Consequently, the CLSM imaging of ZnPc@Gel, while offering some qualitative insights into gel behaviour under mechanical stress, did not reliably capture the morphology of the hydrated gel in its native state. These results show the limitations of using low quantum yield fluorophores like ZnPc for CLSM analysis of supramolecular hydrogels.

#### DPP-BC@Gel

**Figure 3:**
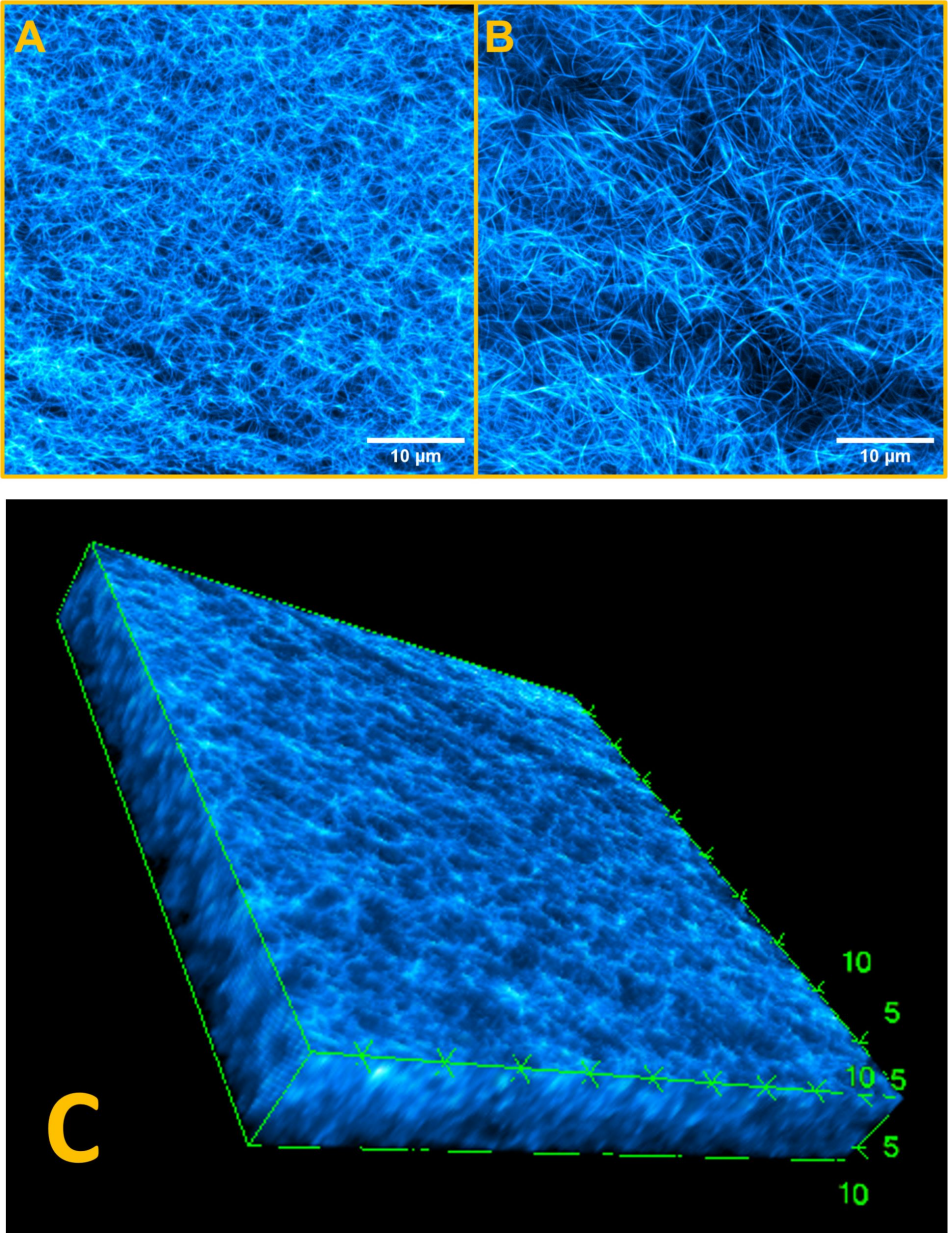
CLSM micrographs of DPP-BC@Gel, [DPP-BC] = 100 µM. (A) Micrograph of the bulk fibre morphology of DPP-BC@Gel. (B) Micrograph of the fibre morphology of DPP-BC@Gel where the gel sample is in contact with the glass coverslip. Imaging details: laser 561 nm, emission range 565–700 nm, laser power 30%. (C) Three-dimensional view of a *z*-stack of *x*–*y* slice images collected of DPP-BC@Gel in an area of high fibre density. Units of boundary boxes are micrometres. Imaging details: laser 561 nm, emission range 565–700 nm, laser power 30%.

In general, DPP dyes are highly fluorescent and photo-stable molecules [28–29] that have been explored as potential bio-imaging fluorescent probes [30–31]. Incorporation of the DPP-BC dye into the hydrogel system resulted in a marked improvement in CLSM imaging quality (Figure 3) compared with the phthalocyanine derivative. The significantly stronger fluorescence emission of the DPP derivatives under moderate laser excitation (a low percentage of the maximum possible irradiance) enabled individual fibrillar structures to be visualized clearly without the need to compress the gel between glass slides, thus preserving the integrity of the hydrated network. This ease of imaging contrasted sharply with the ZnPc-loaded gels, where high laser powers and mechanical compression were necessary to obtain even modest imaging contrast.

The ease of observation of the morphology of the hydrated DPP-BC@Gel sample can be attributed to the higher fluorescence quantum yield of DPP dyes (coupled with their larger extinction coefficient) compared with the phthalocyanine. DPP dyes generally have larger extinction coefficients than zinc phthalocyanines [32–33]. These properties allowed for imaging at lower dye concentrations, minimizing disruption of the native gel morphology by avoiding aggregation or artefacts associated with high fluorophore loadings, an effect previously observed in such systems [26]. As illustrated in Figure 3A,B, the DPP-BC-containing gels exhibited a robust network of interconnected fibres and bundles across the CLSM micrographs, features which were largely indistinct or absent in the images of the ZnPc-loaded gels. Additionally, the uniform fluorescence intensity and low background signal in DPP-BC@Gel samples facilitated the acquisition of three-dimensional (*z*-stack) datasets, providing insight into the fibre architecture and continuity throughout the bulk of the gel (Figure 3C). Such datasets were difficult or impossible to obtain in the case of ZnPc@Gel, where low signal-to-noise ratios and heterogeneous emission limited the depth and clarity of CLSM imaging.

### Cryo-SEM imaging

#### ZnPc@Gel and DPP-BC@Gel

Whereas CLSM relies on fluorescence to visualize hydrated gels directly, cryo-SEM provides an electron-based image of frozen desolvated gels. Rapidly freezing the sample (plunge-freezing in liquid nitrogen slush followed by freeze-fracture) preserves its near-native hydrated structure (usually not the case, often ice crystals form, the presence of the ethanol and/or the mechanical strength of the gel are believed to obviate the problem here). The subsequent sublimation of surface ice exposes the underlying fibres. This allows for high-resolution imaging without the extensive drying that can collapse fibrous networks during the formation of the xerogel in conventional SEM preparation [34]. The study of the gel systems prepared here was potentially complicated by the fact that the bis(imidazolium)-based gels are formed in 1:1 water–ethanol. A high ethanol content posed a potential challenge to effectively freezing the native gel structure. The freezing point of a 1:1 water–ethanol mixture is approximately −40 °C, so less rapid freezing may have been expected resulting in excessive ice crystal formation [35]. It is also possible that the differing melting points of water and ethanol (0 and −114 °C, respectively) could result in phase separation whereby the water freezes, first expelling the ethanol into concentrated domains, which alters microscale material morphology. Such a phenomenon has been exploited for the fractional freezing of water–ethanol mixtures and in liposomal formulations containing water and DMSO [36]. However, using plunge freezing in nitrogen slush, followed by sublimation, effectively preserved gel morphology with minimal observable artefacts.

**Figure 4:**
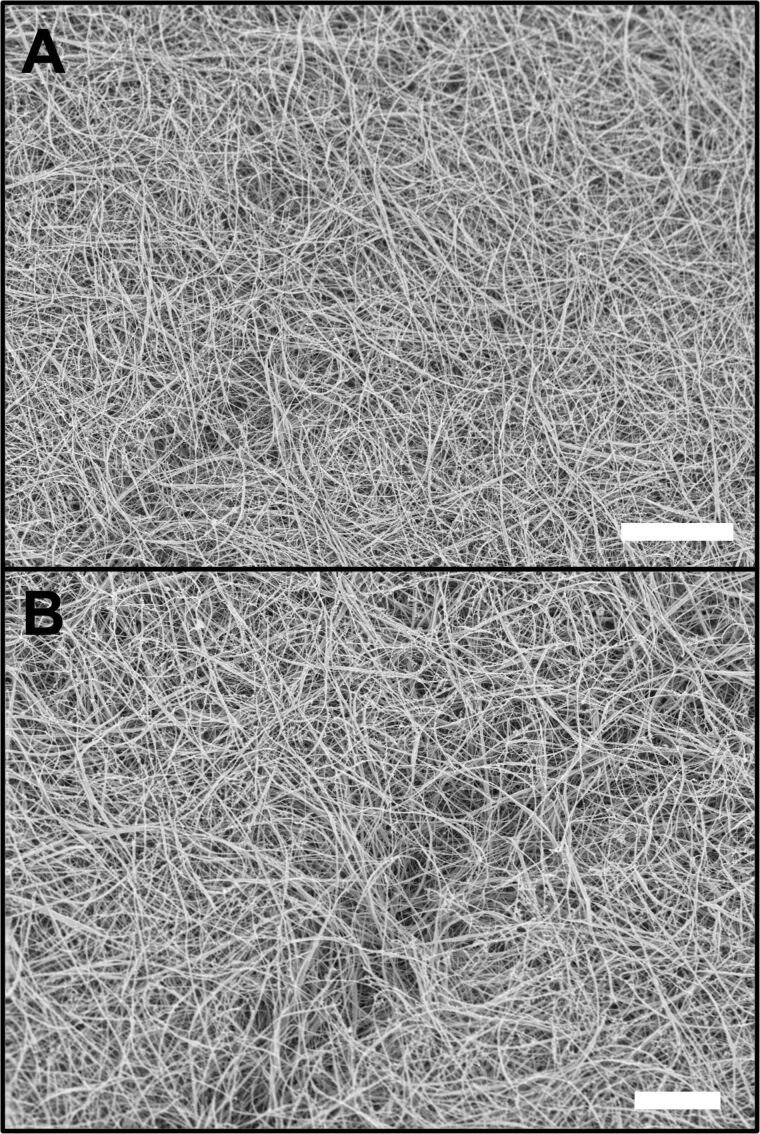
Representative cryo-SEM micrographs of DPP-BC@Gel. Gel prepared in 1:1 water–ethanol and [**1**·2Br] = 12 mM. [DPP-BC] = 150 μM. The scale bar for both images A and B is 10 µm. Accelerating voltage for both images was 3.3 kV (InLens detector).

In DPP-BC-loaded gels (Figure 4), cryo-SEM similarly captured the fibrous supramolecular morphology, often with curved and branching networks that resemble what was directly visualized by CLSM. Here, the correlation between cryo-SEM and CLSM imaging is strong. The fibre outlines, apparent bundling, and overall network architecture are similar in both images, demonstrating that the bright DPP fluorescence accurately reports on the gel’s intrinsic morphology. Minimal compression or sample manipulation is needed for DPP-BC@Gel CLSM imaging, so the morphology seen in CLSM agrees well with the near-native state captured by cryo-SEM.

**Figure 5:**
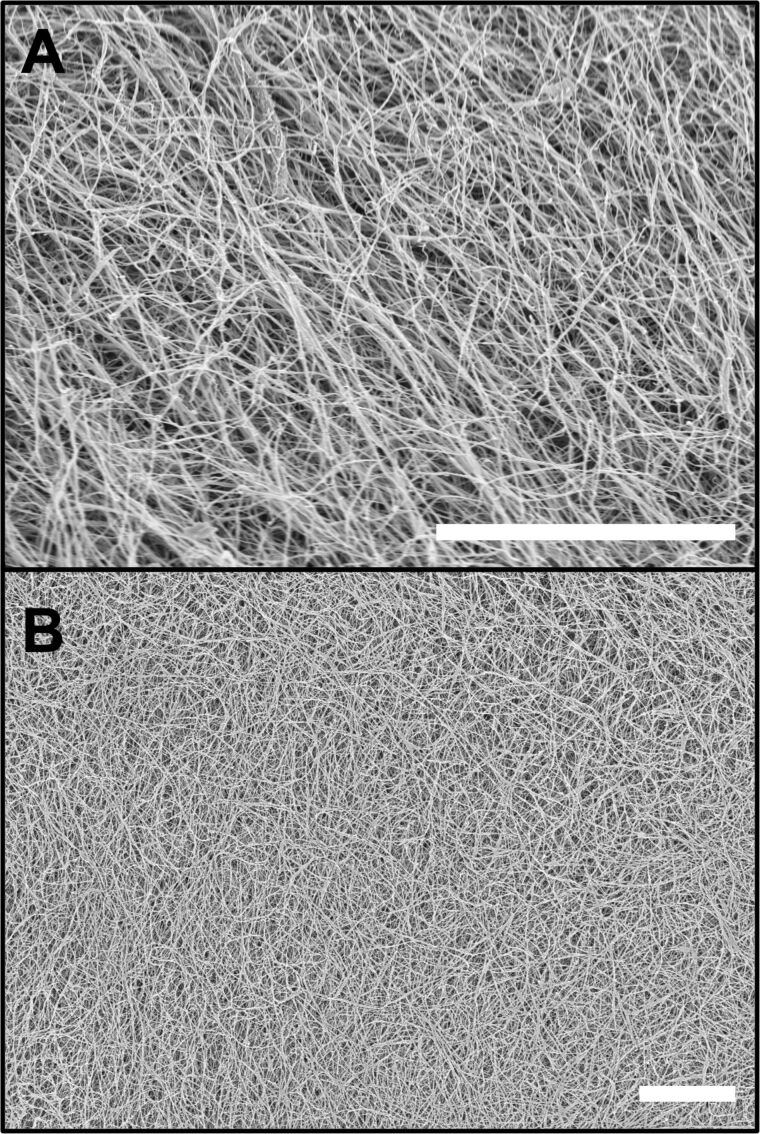
Representative cryo-SEM micrographs of ZnPc@Gel. Gel prepared in 1:1 water–ethanol and [**1**·2Br] = 12 mM. [ZnPc] = 100 μM. The scale bar for both images A and B is 10 µm. Accelerating voltage for both images was 3.3 kV (InLens detector).

Despite ZnPc@Gel looking poorly defined in CLSM images, cryo-SEM micrographs (Figure 5) consistently revealed a dense fibrous network, confirming that the weak fluorescence signal in the CLSM measurements was not as a result of the absence of fibres. Even at modest ZnPc concentrations, the cryo-SEM images show interwoven fibres with typical widths of tens of nanometres. This observation underscores a key limitation in ZnPc-based CLSM imaging: The gel does contain well-developed fibrils, but the dye’s low brightness masks them in CLSM micrographs. Cryo-SEM, by removing the need for sufficient fluorescence emission, effectively confirms the presence and continuity of fibrous bundles that remain undetected under the confocal conditions. Importantly, no material is seen in between the fibres, indicating that the ZnPc is bound to the gelator.

**Figure 6:**
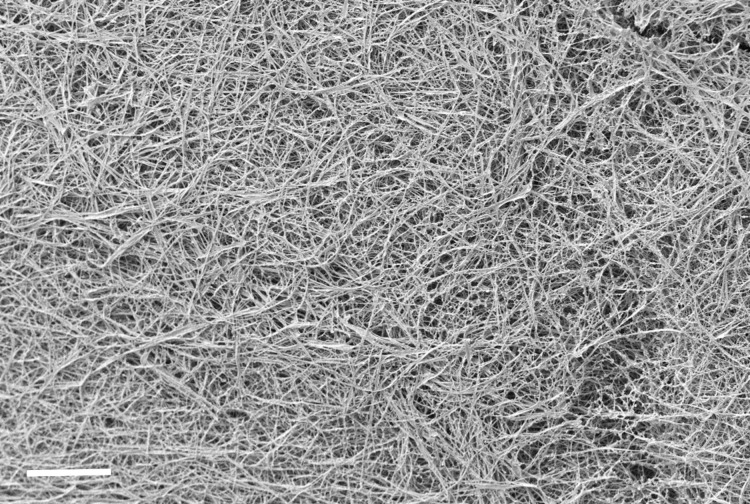
Representative cryo-SEM micrograph of the pure xerogel **1**·2Br (formed at 12 mM in 1:1 water–ethanol prior to cryo-treatment). The scale bar corresponds to 10 µm. Accelerating voltage was 3.3 kV (InLens detector).

There is little observable difference in the gel morphology between the gel containing ZnPc and that containing DPP-BC, with both exhibiting long, curving fibres characteristic of the native **1**·2Br gel morphology [22]. This similarity suggests that (at the concentrations explored in this study), the encapsulated fluorophore does not induce any distinguishable microscale morphological change in the gelator’s fibres. As a control, we also measured the pure gelator **1**·2Br, which has a very similar morphology to those incorporating the fluorophores (Figure 6). Therefore, the organic anions do not seem to affect greatly the gel morphology, while in another case we showed that the anion be important in determining gel morphology at varying concentrations of the fluorophores [26].

### Imaging comparison

**Figure 7:**
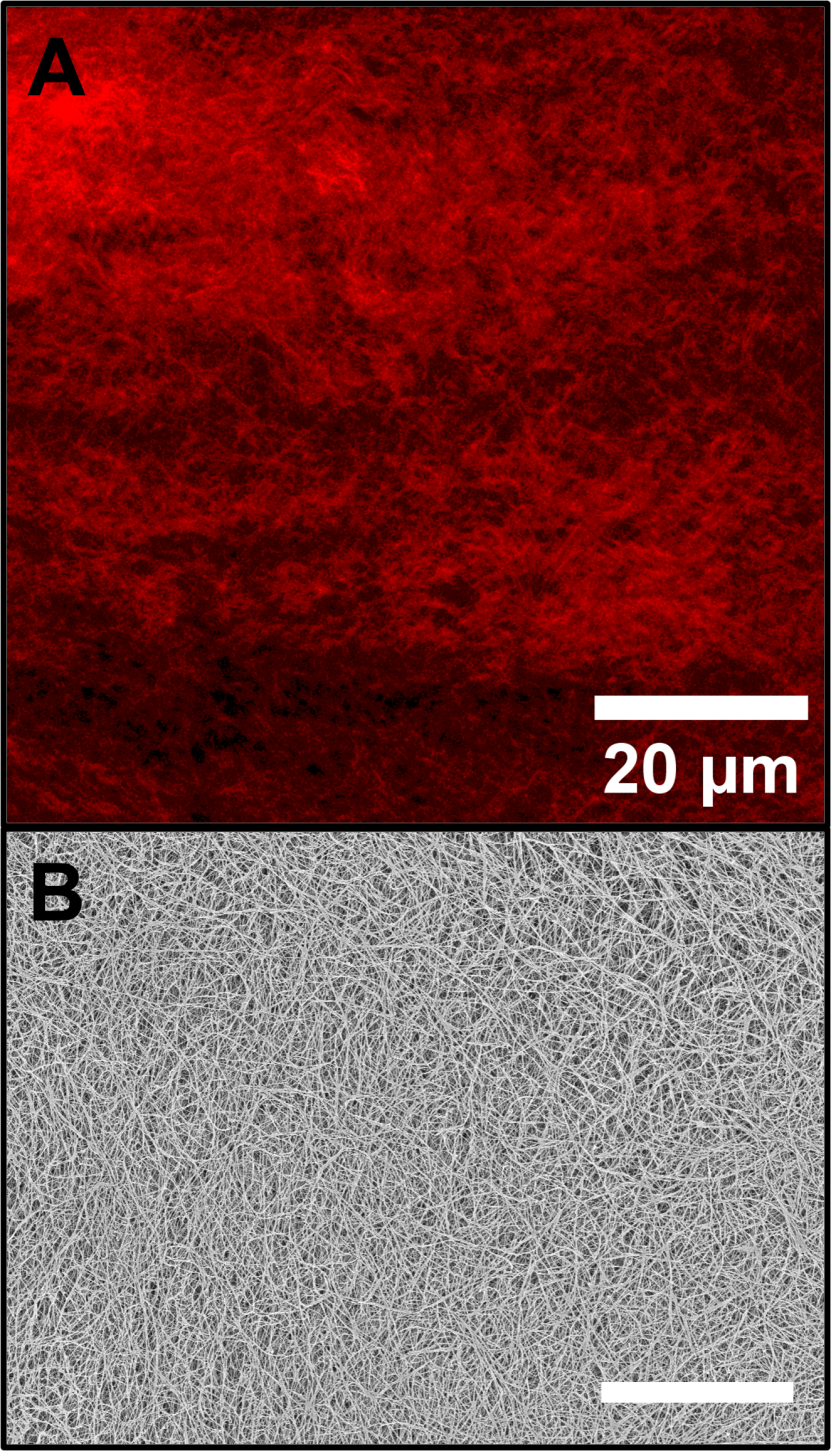
CLSM (A) and cryo-SEM (B) micrographs of ZnPc@Gel. [ZnPc] = 100 μM. Gel prepared in 1:1 water–ethanol and [**1**·2Br] = 12 mM. The scale bar for both images is 20 µm. Accelerating voltage for cryo-SEM was 3.3 kV (InLens detector). CLSM imaging parameters: laser 650 nm, emission LP filter 650 nm, laser power 100%.

A direct comparison of ZnPc@Gel and DPP-BC@Gel imaged using CLSM and cryo-SEM reveals significant differences in the fidelity of morphological features captured by each technique, particularly in relation to the chosen fluorophore. In the case of ZnPc@Gel, CLSM imaging presented significant limitations. Despite relatively high fluorophore loadings, the low fluorescence quantum yield of ZnPc resulted in poor signal-to-noise ratio and relatively unresolved fibre features. Only regions of the gel that were broken or compressed allowed for partial visualization of fibrous domains. This poor sensitivity limited the extent to which CLSM could be reliably used to extract morphological information from ZnPc-containing gels, and no subtle features such as fibre curvature or bundling could be discerned with confidence. Conversely, cryo-SEM imaging of ZnPc@Gel enabled a more comprehensive visualization of gel morphology (Figure 7).

**Figure 8:**
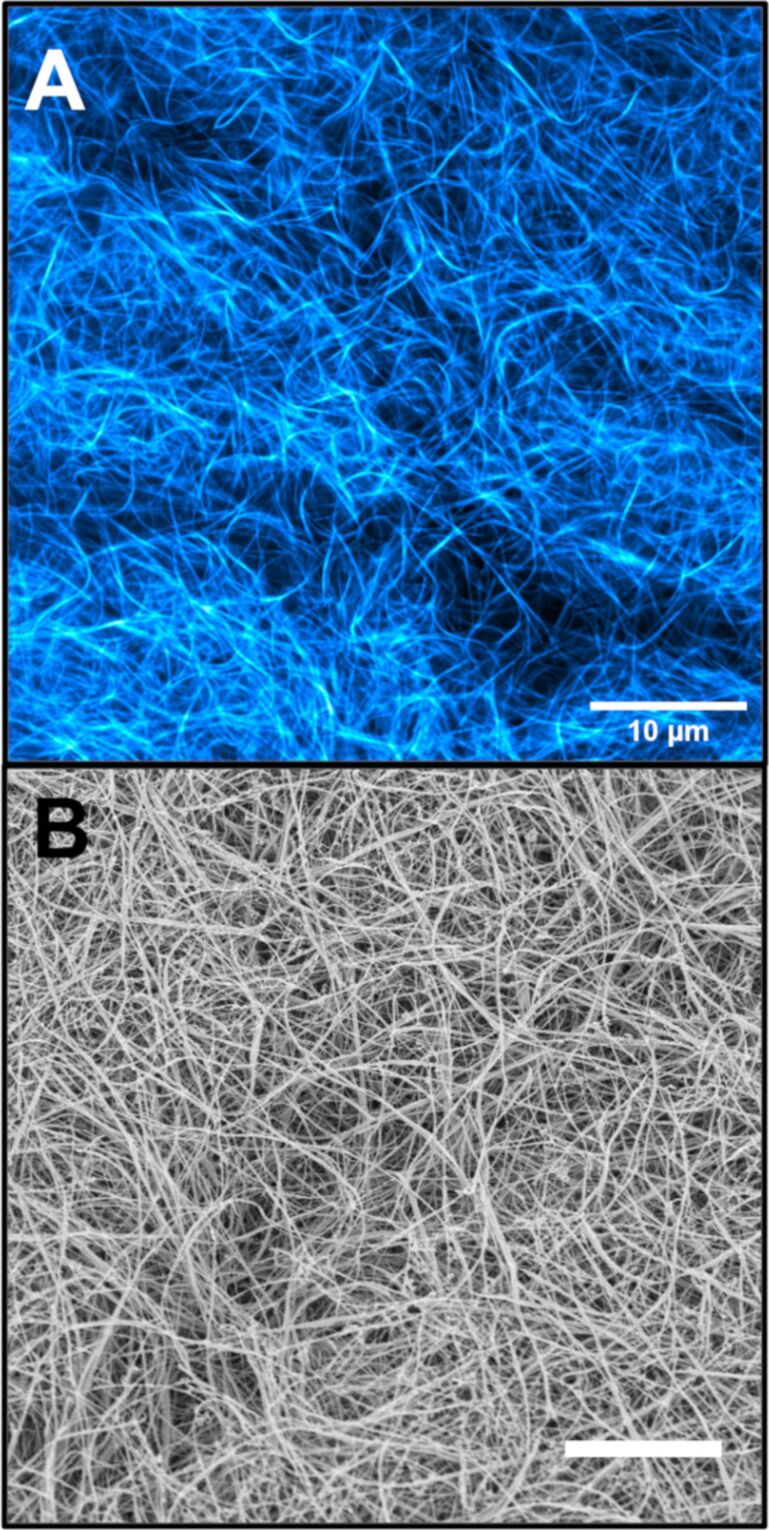
CLSM (A) and cryo-SEM (B) micrographs of DPP-BC@Gel. [DPP-BC] = 150 μM. Gel prepared in 1:1 water–ethanol and [**1**·2Br] = 12 mM. The scale bar for both images is 20 µm. Accelerating voltage for cryo-SEM was 3.3 kV (InLens detector). CLSM imaging details: laser 561 nm, emission range 565–700 nm, laser power 30%.

By contrast, the DPP-BC@Gel displayed a well-defined and highly resolved fibrous morphology using both CLSM and cryo-SEM. The strong fluorescence emission of DPP-BC enabled clear CLSM imaging without the need for sample compression, allowing for visualization of fibre curvature, density gradients, and local structural heterogeneity. Significantly, this morphology closely matched the features observed in cryo-SEM, where fibre curvature and network continuity were again evident (Figure 8). The strict correlation between CLSM and cryo-SEM for DPP-BC@Gel suggests that this fluorophore accurately reflects the native gel structure without significantly disrupting it.

Domains consisting of lower fibre density can also be observed with both imaging techniques. With CLSM alone, it would not be provable whether these low-fibre-density areas were truly free of fibres or simply contained fibres that were not encapsulating fluorophore. Given that the same low-fibre-density areas are seen in cryo-SEM and are of the same approximate size, this structural heterogeneity can be confirmed not to be an artefact of CLSM but rather intrinsic to the microscale morphology of the self-assembled hydrogel.

These differences indicate that the choice of fluorophore can critically impact not only the quality of CLSM imaging but also the interpretation of gel morphology. ZnPc requires high loadings to be visualized by CLSM; yet, even at such concentrations, image clarity is poor, and the gel may be structurally perturbed. In contrast, DPP-BC enables high-resolution imaging across both techniques with minimal structural disruption. This divergence underscores the importance of selecting fluorophores not merely for brightness, but also for compatibility with the gel matrix and the imaging modality in question.

## Conclusion

This study highlights the strengths of CLSM and cryo-SEM in characterizing the morphology of imidazolium-based supramolecular hydrogels. The techniques are complementary to, for example, cryogenic transmission electron microscopy where submolecular detail can be determined [36].

The choice of fluorophore proved critical to the effectiveness of CLSM imaging. DPP-BC, a highly fluorescent and water-soluble DPP derivative, enabled detailed visualization of the fibrous gel architecture in its hydrated state without the need for sample compression or high excitation intensities. These CLSM images closely aligned with cryo-SEM micrographs of the same material, confirming that DPP-BC accurately reflects native gel morphology, and that cryo-SEM treatment does not disturb the fibre network.

In contrast, ZnPc@Gel presented significant challenges under CLSM because of the relatively low absorbance and fluorescence quantum yield of the phthalocyanine dye, necessitating high fluorophore concentrations and mechanical compression for imaging. These constraints led to artefacts and obscured the true morphology of the gel. However, cryo-SEM imaging of ZnPc@Gel revealed well-formed fibrous networks similar to those seen in DPP-BC@Gel, demonstrating that the poor CLSM performance of ZnPc arises from photophysical limitations rather than morphological differences.

Together, these findings reveal the value of combining CLSM and cryo-SEM (where alternative sample preparation techniques exist [37–38]) for the morphological analysis of soft materials, particularly where fluorophore properties or sample preparation requirements might obscure key features in a single modality. Moreover, the results stress the importance of fluorophore selection in CLSM studies of supramolecular systems, especially when accurate structural correlation to the native gel state is desired.

## Experimental

### Materials

Zinc phthalocyanine tetrasulfonic acid (ZnPc) was purchased from PorphyChem. Preparation of the dicationic amphiphile gelator (**1**·2Br) was carried out through an established synthetic procedure [39]. DPP-BC was prepared using a reported method [40].

### Preparation of fluorophore@Gels

To prepare a gel sample incorporating a water-soluble fluorophore, an aqueous fluorophore solution of the desired concentration was prepared from a 2.4 mM stock solution and was transferred and mixed with an equal volume of an ethanolic **1**·2Br solution (24 mM) in a glass vial. An equal volume of de-ionised water was then added to this and mixed, allowing for gel formation. The final fluorophore concentration in the gels was varied from 50 to 150 μM. The final **1**·2Br gelator concentration was always 12 mM. Turbidity was observed upon mixing aqueous and ethanolic solutions, and gelation generally occurred in less than one minute (as judged by vial inversion) to yield the gel materials containing the chosen fluorophore.

### Photophysical characterization of fluorophore@Gels

UV–visible absorption spectroscopy was performed using a Cary 5000 UV–visible spectrophotometer (Agilent). Fluorescence spectroscopy and absolute fluorescence quantum yield measurements (given in Supporting Information File 1) were carried out on a FLS 980 spectrometer (Edinburgh Instruments) equipped with a front face sample holder or integrating sphere. The fluorescence and fluorescence quantum yield measurements were carried out using quartz cuvettes of 1 and 10 mm path lengths, respectively.

### Confocal laser scanning microscopy imaging

CLSM imaging was carried out on a Zeiss LSM 900 with AiryScan 2 detector (essential for observation here) on an Observer platform system running Zen Blue software. The scanning was in unidirectional sequential line mode with sixteen times averaging to reduce noise. Images were analysed with ImageJ. Objectives used during this study were: Fluar 5× (N.A. = 0.25), resolution 1.33 µm, total magnification 50×, bright field condenser. Plan Apochromat 20× (N.A. = 0.8) (DICII), resolution 420 nm, total magnification 200×. LD Plan-Neofluar 40× (N.A. = 0.6) (Ph2 DICII), resolution 560 nm, total magnification 400×. C-Apochromat 63× (N.A. = 1.2 W) (Water DICII), resolution 280 nm, total magnification 630×. EC Plan-Neofluar 100× (N.A. = 1.3 Oil) (DIC), resolution 140 nm, total magnification 1000×. Spectral irradiance at 561 nm and 100% laser power is 88.4 W·m^−2^.

For imaging of compressed samples, a sample of gel around the size of the end of a spatula was placed upon a circular glass coverslip in a holder. A plastic O-ring was placed on the glass coverslip around the sample and another circular glass plate was placed on top of this. The holder was then screwed, which compressed the gel between the two glass plates to form a thin section of gel. This sample was then viewed under the Zeiss LSM 900 in bright-field mode, before focusing on a bubble or other feature of gel under the Fluar 5× objective, the Plan Apochromat 20× objective was then used to focus further on the chosen feature. When focused on the sample feature, the 63×/100× immersion objectives were coated with oil/water–oil and focused until some fibre morphology could be seen. From here, a suitable excitation laser and filters were activated and focusing continued until sample morphology could be seen in confocal Airyscan mode. For imaging of uncompressed samples, the same procedure as above was followed but the gel sample placed upon the glass coverslip was not compressed with another glass plate.

### Cryo-SEM imaging

Cryo-SEM was carried out using a Zeiss Crossbeam 550 fitted with a Quorum 3010T cryo-stage and preparation chamber. Samples were prepared for analysis by freezing in liquid nitrogen slush on grooved cylindrical aluminium stubs, using rivets for freeze fracture, with a Quorum PP3010 Prepdek slush freezer. The stubs were then secured onto the sample shuttle of the cryo-system before transfer to the preparation station at −170 °C. Freeze fracture was carried out by removing the top half of the rivet with the fracturing knife attached to the PP3010.

The gel sample underwent 5 min of etching via sublimation at −100 °C followed by sputtering in an argon environment using platinum for 60 s at a current of 10 mA. Once coated, the shuttle was transferred to the cryo-stage in the SEM chamber and maintained at −170 °C. Finally, the microstructure of the hydrogel was then imaged by SEM at an accelerating voltage of 3.3 kV using the SESI and InLens detectors.

## Supporting Information

File 1 Additional figures and tables.

File 2 CLSM Z-stack animation video of DPP-BC (low fibre density) over the course of 42 frames.

File 3 CLSM Z-stack animation video of DPP-BC (high fibre density) over the course of 29 frames.

## Data Availability

All data that supports the findings of this study is available in the published article and/or the supporting information of this article.
