# Supplementary material for: Visualizing nanostructures in supramolecular hydrogels: a correlative study using confocal and cryogenic scanning electron microscopy
Source: Beilstein J Nanotechnol. 2025 Dec 12;16:2274–84. doi: 10.3762/bjnano.16.156 (PMC12706373; doi:10.3762/bjnano.16.156)
Supplement: File 1 — Additional figures and tables. [file Beilstein_J_Nanotechnol-16-2274-s001.pdf]

BEILSTEIN JOURNAL OF NANOTECHNOLOGY

Supporting Information

for

Visualizing nanostructures in supramolecular hydrogels: a correlative study using confocal and cryogenic scanning electron microscopy

Shaun M. Smith, Ferdinando Malagreca, Jacqueline Hicks, Giuseppe Mantovani, David B. Amabilino, Christopher Parmenter and Lluïsa Pérez-García

Beilstein J. Nanotechnol. **2025,** *16,* 2274–2284. doi:10.3762/bjnano.16.156

Additional figures and tables

License and Terms: This is a supporting information file under the terms of the Creative Commons Attribution License (https://creativecommons.org/licenses/by/4.0). Please note that the reuse, redistribution and reproduction in particular requires that the author(s) and source are credited and that individual graphics may be subject to special legal provisions.
The license is subject to the *Beilstein Journal of Nanotechnology* terms and conditions: (https://www.beilstein-journals.org/bjnano/terms)

Table of Contents

1. Supplementary Figures

2. Supplementary Table

1. Supplementary Figures

Figure S1

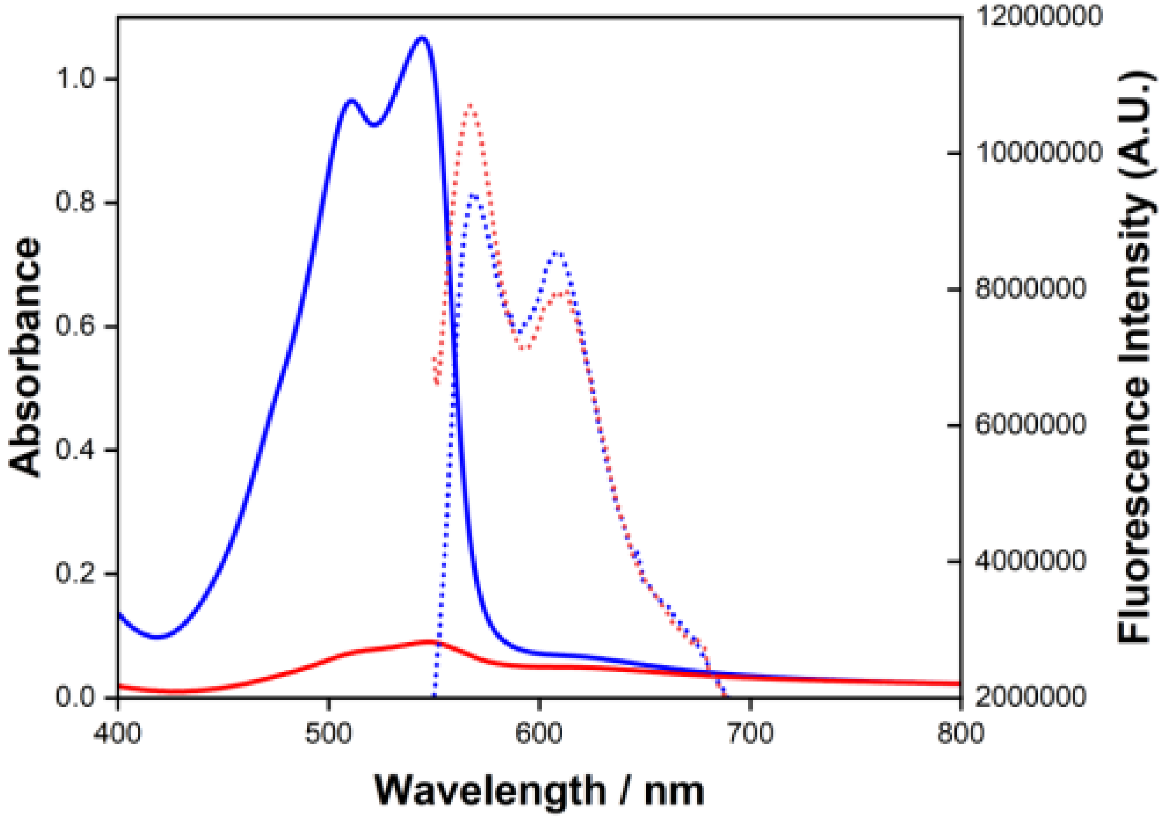

Figure S1: Plot displaying the absorption spectrum of DPP-BC in 1:1 water-ethanol solution, [DPP-BC] = 100 µM (blue solid line), absorption spectrum of DPP-BC@Gel, [DPP-BC] = 200 µM (red solid line), fluorescence emission spectrum of DPP-BC in 1:1 water-ethanol, [DPP-BC] = 100 µM (blue dotted line), and fluorescence emission spectrum of DPP-BC@Gel, [DPP-BC] = 200 µM (red dotted line). λ_{exc} = 530 nm for both fluorescence emission measurements.

Figure S2

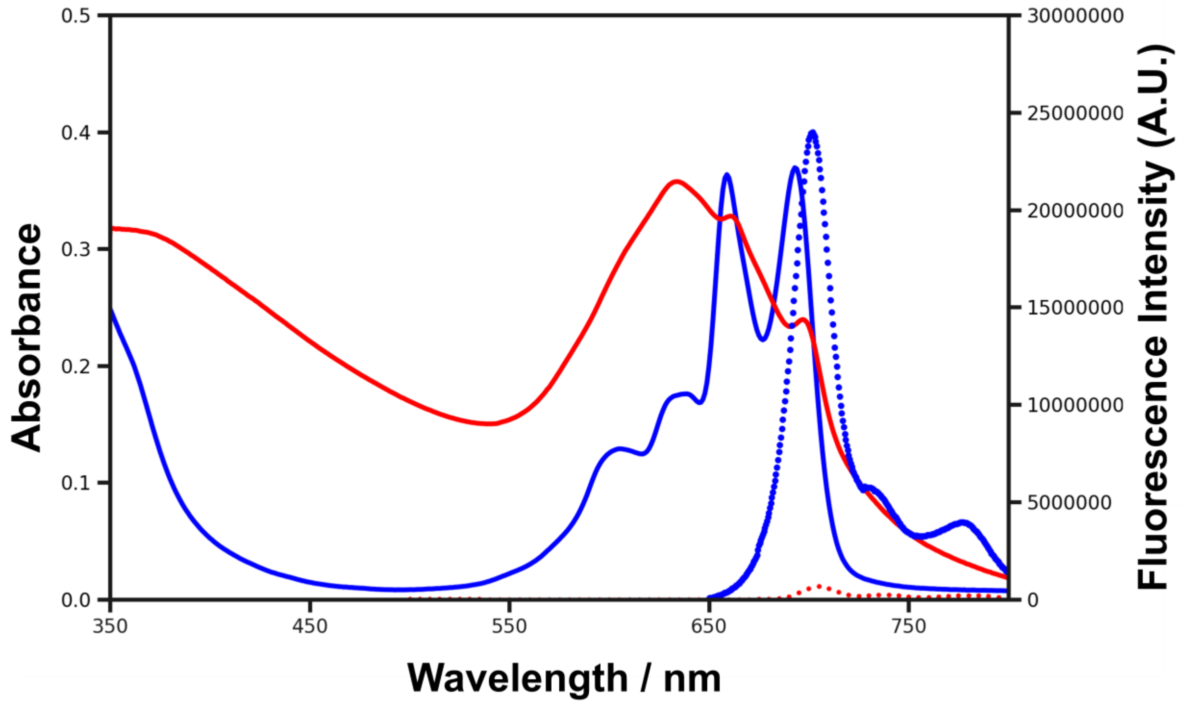

Figure S2: Plot displaying the absorption spectrum of ZnPc in 1:1 water-ethanol solution, [ZnPc] = 10 µM (blue solid line), absorption spectrum of ZnPc@Gel, [ZnPc] = 200 µM (red solid line), fluorescence emission spectrum of ZnPc in 1:1 water-ethanol, [ZnPc] = 20 µM (blue dotted line), and fluorescence emission spectrum of ZnPc@Gel, [ZnPc] = 100 µM (red dotted line). λ_{exc} = 625 nm for both fluorescence emission measurements.

2. Supplementary Table

Table S1: Experimentally determined quantum yield of fluorescence values for ZnPc and DPP-BC in 1:1 water-ethanol solutions.

Compound	Φ_F
Zinc phthalocyanine tetrasulfonic acid (ZnPc)	0.07
DPP-BC	0.84
