# Supplementary figures and images for: Visualizing nanostructures in supramolecular hydrogels: a correlative study using confocal and cryogenic scanning electron microscopy
Source: Beilstein J Nanotechnol. 2025 Dec 12;16:2274–84. doi: 10.3762/bjnano.16.156 (PMC12706373; doi:10.3762/bjnano.16.156)

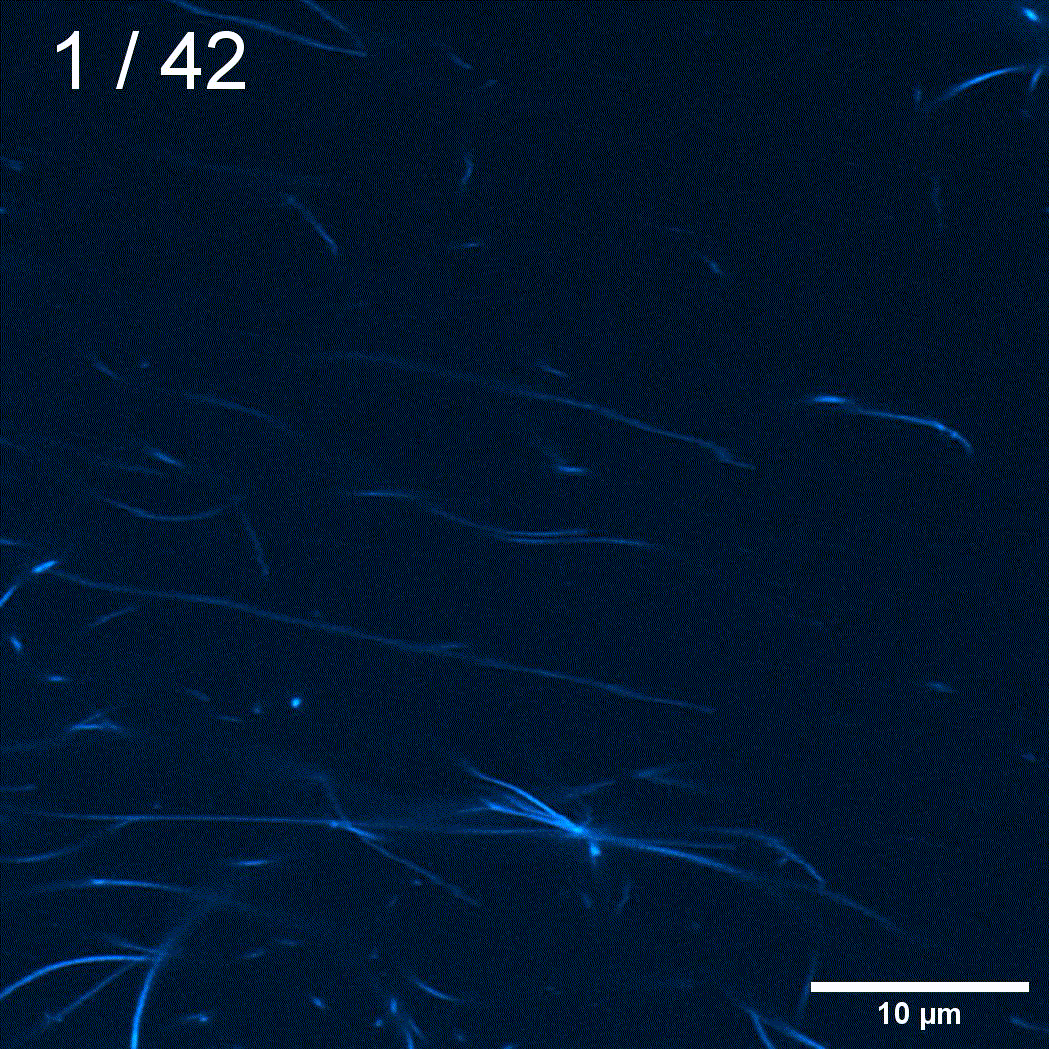

Supplement: File 2 — CLSM Z-stack animation video of DPP-BC (low fibre density) over the course of 42 frames. [file Beilstein_J_Nanotechnol-16-2274-s002.gif]

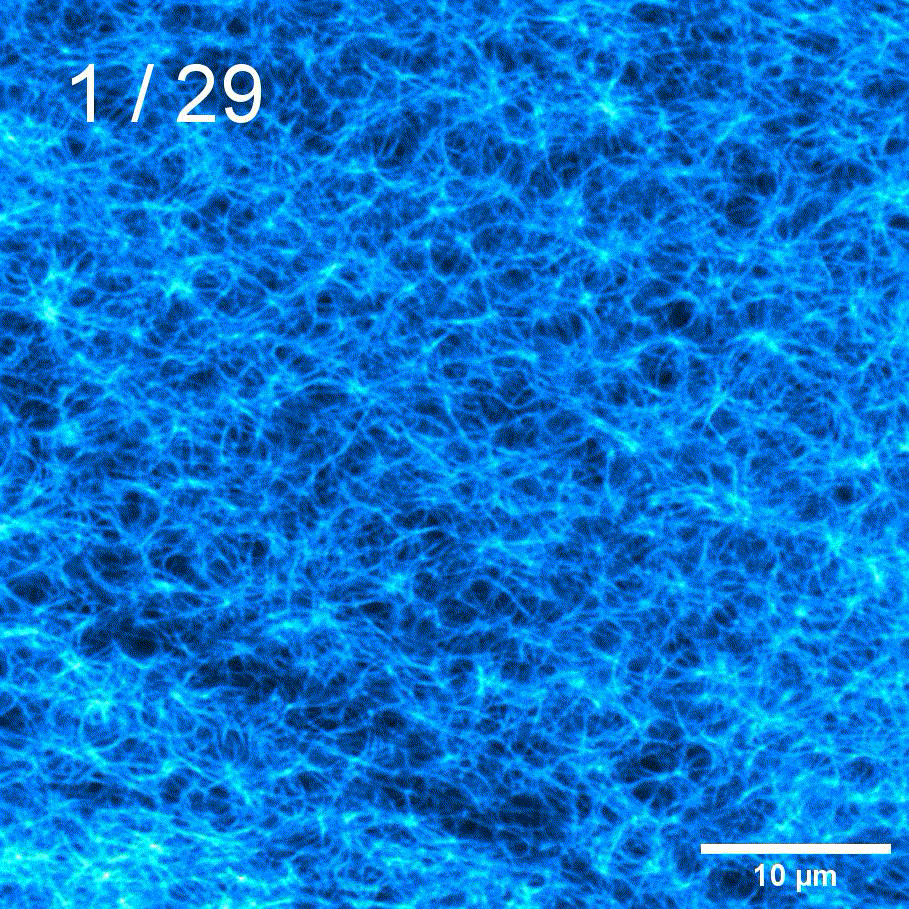

Supplement: File 3 — CLSM Z-stack animation video of DPP-BC (high fibre density) over the course of 29 frames. [file Beilstein_J_Nanotechnol-16-2274-s003.gif]
